# Differential relationships of family drinking with alcohol expectancy among urban school children

**DOI:** 10.1186/1471-2458-11-87

**Published:** 2011-02-08

**Authors:** Chuan-Yu Chen, Carla L Storr, Chieh-Yu Liu, Kuang-Hung Chen, Wei J Chen, Keh-Ming Lin

**Affiliations:** 1 Division of Mental Health and Addiction Medicine, Institutes of Population Health Sciences, National Health Research Institutes, Miaoli County, Taiwan; 2 Institute of Public Health, National Yang-Ming University, Taipei, Taiwan; 3 Department of Public Health, National Taiwan University, Taipei, Taiwan; 4 School of Nursing, University of Maryland, Baltimore, Maryland, USA; 5 Department of Nursing, National Taipei College of Nursing, Taipei, Taiwan; 6 Institute of Epidemiology, College of Public Health, National Taiwan University, Taipei, Taiwan

## Abstract

**Background:**

Positive alcohol outcome expectancy has consistently been linked with problematic drinking, but there is little population-based evidence on its role on early stages of drinking in childhood. The present study seeks to understand the extent to which drinking of family members is differentially associated with the endorsement of alcohol expectancy in late childhood.

**Methods:**

A representative sample of 4th and 6th graders (N = 2455) drawn from 28 public schools in an urban region of Taiwan completed a self-administered paper-and-pencil questionnaire. Each student provided information on alcohol expectancy, drinking experiences, and individual and family attributes. Complex survey analyses were performed to evaluate the relationship, with stratification by children's alcohol drinking history.

**Results:**

An estimated 29% of the 4^th ^graders and 43% of the 6^th ^graders had initiated alcohol consumption (over 40% of them had drank on three or more occasions). Alcohol drinking-related differences appear in both the endorsement and the correlates of alcohol expectancy. Positive alcohol expectancy was strongly associated with family drinking, particularly the dimension of "enhanced social behaviors"; negative alcohol expectancy was inversely associated with drinking frequency. Among alcohol naïve children, significant connections appear between paternal drinking and three dimensions of positive alcohol expectancy (i.e., enhanced social behaviors:β_wt _= 0.15, promoting relaxation or tension reduction:β_wt _= 0.18, and global positive transformation:β_wt _= 0.22).

**Conclusions:**

Individual tailored strategies that address family influences on alcohol expectancy may be needed in prevention programs targeting drinking behaviors in children.

## Background

Alcohol related problems are a huge burden on national productivity in diverse populations and have taken an increasingly heavy toll on health globally [1,2]. Of particular note, alcohol-related problems seem to disproportionately affect youth and young adult populations [3,4]; regardless of region and gender, individuals between the ages of 15-29 years consistently have the highest proportion of alcohol-attributable deaths [5]. Moreover, a growing body of empirical literature suggests that alcohol use in childhood may not only increase the risks for a range of health problems, but also poses harm to long-term development and wellbeing [6,7]. Therefore, in order to provide age-tailored preventive programs for children it is important to understand the factors that can shape the earliest experiences of alcohol involvement in a developmental framework [8-10].

Alcohol expectancies involve the learning and memory processes that link reinforcement information to the use of alcohol and to the various contexts in which alcohol is usually consumed. Previous observations of children and adolescents suggest there is a gradual shift from negative (aversive) to positive (rewarding) alcohol expectancies as one becomes older [11-14]. Etiologic and treatment researchers are increasingly finding that alcohol expectancy has several dimensions and plays a significant role in the progression of initiation, heavy use, abuse or dependence, and relapse among adolescent and adult populations [15-18]; generally, heavy or problematic drinkers are more likely to endorse higher positive alcohol expectancy. To illustrate, Christiansen and colleagues reported that high scores in two dimensions (i.e., "Alcohol can enhance social behaviors" and "Alcohol improves cognitive and motor functions") significantly predicted the risk of increased consumption (i.e., quantity/frequency) and problem drinking a year later among junior high school students in Detroit [19]. A prospective study involving a sample of 6^th ^graders in one suburban Maryland school district found that children with higher drinking expectancies were twice as likely to initiate drinking and that the risks were even greater among children with low parental expectation [20]. Another longitudinal study of 8-10 year old elementary school-attending girls in Pittsburgh also indicated that, with adjustment for race/ethnicity groups, family structure, parental drinking, and personal alcohol use, girls with the highest negative expectancy scores had a lower likelihood of reporting future intention of alcohol use [21].

Prior research has revealed several aspects of social environment that may influence the natural history of alcohol use problems in younger populations [6,7,22-24]. It is believed that alcohol expectancies can be acquired either directly by the drinking of alcohol or indirectly by observing the drinking behaviors of others, and the literature reports the identification of a variety of factors that may be associated with the endorsement of alcohol outcome expectancy in younger populations, including individual characteristics such as age and gender; social influences such as parental and peer drinking [11-13,25]. Parental drinking behaviors have been consistently identified as strong predictors for offspring alcohol use in adolescence, and are posited to operate via processes that include behavioral modeling and increased alcohol availability at home [24,26,27]. Building upon a family-based prospective study in the Netherlands, van der Vorst and colleagues found parental drinking was inversely associated with having strict alcohol-specific rules, which may have subsequently delayed early initiation of alcohol among the adolescents [28]. Following up the offspring of parents with alcohol use disorders in the U.S., Shen and colleagues found the links between familial risk and subsequent manifestation of drinking behaviors (e.g., frequency, quantity per occasion, and physical consequences) in adolescents may possibly be mediated by offspring' alcohol expectancy, in particular dimensions such as enhanced social behaviors and increased arousal [29].

Population-based observational studies of children [21,30] and studies of high-risk youth (e.g., offspring of alcoholics)[17,29,31,32] have been accumulating evidence on potential correlates of alcohol expectancy. Despite recognizing racial/ethnic variation in the associations between alcohol expectancy and alcohol involvement [33,34], very few studies have been done on children growing up in a non-Western culture. Considering possible heterogeneity in norms, availability, and policies toward underage drinking, the investigation of the correlates or predictors for alcohol expectation in different cultures may increase our understanding of the extent to which social or cultural contexts may shape the emergence and impact of alcohol expectations during childhood and adolescence. Furthermore, previous investigations on alcohol outcome expectancy in children have often relied on a unidimensional measure [20], and it remains unclear whether the relationship with individual or contextual attributes may vary across dimensions of alcohol expectancy.

Being an integral part of the culture of Taiwan, alcoholic beverages have long been considered as an indispensable element in promoting cheerfulness and drinking facilitates interactions in family reunions or other types of gatherings. In such a cultural context, most Taiwanese have their first chance to drink alcohol at a family occasion in early childhood [35]. Extending prior research, the aims of this study are to understand how family characteristics (e.g., family drinking) may account for the endorsement of several dimensions of alcohol expectancy in primary school-age Taiwanese children.

## Methods

### Participants and sampling procedures

The present research was derived from the baseline data of the Alcohol-Related Experiences among Children (AREC), a study designed to examine determinants of alcohol-related experiences from childhood to adolescence in Taiwan. In brief, the study involves multi-stage probability sampling which began with a complete list of public elementary schools (n = 141) in an urban region in the 2006-07 year. In order to produce a representative balance of students, we randomly selected 28 schools from four strata defined by school administrative and neighboring characteristics. Next, three classes were randomly selected from both 4^th ^grade (~age 10) and 6^th ^grade (~age 12) within each of selected schools, and all students were selected for schools with three or less classes in the designated grades. All of the students in the sampled classrooms were eligible to participate in this study. Detailed information on the study sample and sampling procedure has been described elsewhere [36].

A letter from the National Health Research Institutes (NHRI) was sent to the designated school principals and classroom teachers. When a school-level refusal occurred, a replacement school within the same sampling stratum was randomly selected, and for classroom-level refusals, another classroom within the same school was randomly selected. One week prior to the designated day of assessment, introductory letters and informed consent forms were distributed to the students to deliver to their parents/primary care givers. Only students with "active parental consent" took part in the survey; data were double keyed-in and linked on the basis of individual identification numbers instead of names. A total of 1306 4^th ^and 1324 6^th ^graders completed the questionnaires, yielding the overall class-level response rate was 98.0% and the individual-level response rate was 59.1% for 4^th ^grade and 62.2% for 6^th ^grade.

### Data collection

Pilot testing with students from four elementary schools (4^th ^and 6^th ^graders, n = 210) led to standardization of the survey procedures. Trained assessors started with engagement exercises to promote trust and rapport and to answer questions about confidentiality, followed by a statement that allowed individual students to decline to participate. Next, the assessor read out main sections on the questionnaires, in a manner that sought to overcome inter-individual variations in reading skills and cognitive development. Meanwhile, a co-assessor served as a monitor to help maintain order and to answer individualized questions. In closing, the assessors collected the completed questionnaires to help promote confidentiality and data quality. On average, children required approximately 30 minutes to complete the paper-and-pencil questionnaire. After the questionnaires were collected, all the data were processed via double-entry verification. This study has been approved by National Health Research Institutes Research Ethics Committee (EC 0951104).

### Measures

Family attributes: Parental drinking behaviors were assessed by two items: "*Have you ever seen your father drinking?*" and "*Have you ever seen your mother drinking?*" Sibling drinking was coded as positive if the young child had at least one older brother or sister who had been seen drinking. Monthly allowance was considered as a proxy measure for family socioeconomic status and afterschool adult supervision was evaluated by the attendance of after school program. Two items "*Is your father currently holding a job?*" and "*Is your mother currently holding a job?*" were adopted to assess parental employment status. Family members (e.g., father, mother, and siblings) who currently live together with the participant were also inquired.

Individual attributes: With respect to alcohol drinking behaviors, lifetime experiences were determined by "*Not including a sip of alcohol and alcoholic beverages added in meals, have you ever drunk alcohol in your lifetime*?" followed by an array of questions pertaining to the age of onset of drinking and cumulative frequency of alcohol consumption for those who had initiated alcohol prior to the assessment. Children who have initiated drinking alcohol will be referred to as alcohol experienced vs. alcohol naïve for children who hadn't initiated use by the time of assessment.

Alcohol expectancies were assessed via the Chinese version for Alcohol Expectancy Questionnaire-Children form (CAEQ-C), translated and adapted from the Alcohol Expectancy Questionnaire-Adolescent form (AEQ-A)[37]. Expert reviews and focus groups determined items were appropriate for the reading and literacy skills of the 3^rd ^grade. Seven items in the scale of "alcohol enhances sexuality" (e.g., "*alcohol makes sexual experiences easier and more enjoyable"*) were dropped because the content was deemed inappropriate. Poor loading items were also not included (e.g., "*people drive better after a few drinks of alcohol*," "*a person may have a few drinks of alcohol in order to be part of the group*"). Six dimensions were identified in the preliminary analyses of the 74 binary CAEQ items. However, the present analyses will focus only on the four scales with high reliability (Cronbach alpha greater than 0.6), three representing positive and one representing negative alcohol expectancies. The internal reliability of the scales tended to be higher among older children: "global positive transformation" [GPT], 13 items; Cronbach alphas = 0.78 and 0.81 for 4^th ^and 6^th ^graders respectively, "enhanced or impeded social behaviors" [ESB], 14 items; Cronbach alphas = 0.61 and 0.69 for 4^th ^and 6^th ^graders respectively, "promoting relaxation or tension reduction" [PRTR], 13 items; Cronbach alphas = 0.84 and 0.86 for 4^th ^and 6^th ^graders respectively, and "deteriorated cognitive and behavioral functions" [DCBF], 21 items; Cronbach alphas = 0.83 and 0.86 for 4^th ^and 6^th ^graders respectively. Reliability estimates were also slightly higher among the children who had drank alcohol (alcohol experienced vs. alcohol naïve Cronbach alpha estimates: GPT = 0.81 vs. 0.78, ESB = 0.67 vs. 0.61, PRTR = 0.86 vs. 0.85, and DCBF = 0.86 vs. 0.83). After excluding 103 (7.9%) 4^th ^graders and 72 (5.4%) 6^th ^graders who had illogical responses in either one of three non-AEQ items, the final analytic sample contained 1203 4^th ^graders and 1252 6^th ^graders.

### Statistical analysis

Due to the multistage sampling procedures employed in the present study, we used standard survey analysis procedures to (i) inspect the distribution of individual and family attributes and (ii) to summarize dimension (scale)-specific estimates of alcohol expectancy scores in relation to alcohol experiences. These procedures take into account the sampling strata, sampling weights, and primary sampling unit (i.e., school). In STATA, for the contingency table analyses and linear regression, the survey commands utilize Taylor series linearization to estimate the variance [38]. Concerning alcohol drinking-related differences in the organization of alcohol expectancy information in childhood [12], we will stratify by a child's prior alcohol drinking history before performing multiple linear regression models that will estimate the connection between family attributes and dimension-specific alcohol expectancies.

For variables collected in the forms of categorical response (e.g., living with parents, parental employment, and monthly allowance) in family attributes, given the numbers in some subgroups were too small, we decide to dichotomize them in order to provide precise estimates of relationships. As to alcohol experience measured in continuous (i.e., age at first use) and ordinal (i.e., cumulative occasions of drinking) variables, the dichotomization approach was used in regression analyses with an attempt to avoid the violation of linear correlation assumption (i.e., the age-related related increase in alcohol expectation is not linear from age 3 to 12) and to reflect differential stage of alcohol involvement. Early alcohol initiation (defined by having the first drink before the age of 7) and cumulative consumption of alcoholic beverages (occasional vs. experimental use) were all taken into account when running models on the alcohol experienced children. In this series of analyses, standardized scores were used in order to compare the magnitude of relationship across the four dimensions of alcohol expectation. All the analyses were conducted using STATA Release 9 (College Station, TX: StataCorp LP).

## Results

Table 1 summarizes the family and individual attributes for alcohol naïve and alcohol experienced children. In general, alcohol-experienced children were more likely to be older (i.e., 6^th ^grade), receive larger monthly allowances, and have more family members (i.e., parents or older siblings) engaged in drinking as compared with their alcohol-naïve counterparts. Roughly one fifth of alcohol-experienced children had their first drink before the age of seven and one half had drank on three or more occasions prior to the time of assessment.

**Table 1:** Characteristics of school-attending children by prior alcohol drinking.

**Characteristics**^**a**^	Alcohol naive	Alcohol experienced
	**1577 (%**_**wt**_**)**	**878 (%**_**wt**_**)**
Grade***		
4^th^	858 (54.3)	345 (39.3)
6^th^	719 (45.7)	533 (60.7)
Gender		
Female	806 (50.7)	422 (48.3)
Male	771 (49.3)	456 (51.8)
Living with parents*		
Living with none or one parent	162 (10.0)	124 (14.0)
Living with both parents	1415 (90.0)	754 (86.0)
Parental employment		
None or one employed	448 (27.1)	218 (24.7)
Both employed	1121 (72.5)	655 (74.8)
Monthly allowance (NT^b^)**		
0	525 (33.7)	231 (26.1)
1-499	787 (49.7)	442 (50.7)
500 or above	216 (13.4)	171 (19.4)
After school program attendance		
No	331 (19.4)	193 (20.8)
Yes	1238 (80.0)	676 (78.3)
Paternal drinking***		
No	552 (35.0)	174 (19.9)
Yes	1025 (65.0)	704 (80.1)
Maternal drinking***		
No	978 (62.1)	308 (35.0)
Yes	599 (38.0)	570 (65.0)
Elder sibling's drinking***		
No	709 (44.4)	229 (26.6)
Yes	93 (5.9)	214 (24.4)
NA	775 (49.7)	435 (49.0)
Age at first drink^a,c^		
7 years or older	na	658 (74.1)
Under the age of 7		156 (18.2)
Cumulative frequency of drinking^a,c^		
Experimental use (1 or 2 occasions)	na	466 (52.1)
Occasional use (3 or more occasions)		393 (45.7)

The Alcohol Related Experiences in Childhood (AREC), 2006-2007 (N = 2455).

* p < 0.05; ** p < 0.01; *** p < 0.001.

a. Some columns do not add up to 100% because of missingness.

b. NT: New Taiwan dollars.

c. The denominator involves the children who have had alcohol at least once in their lifetimes.

**Figure 1:**
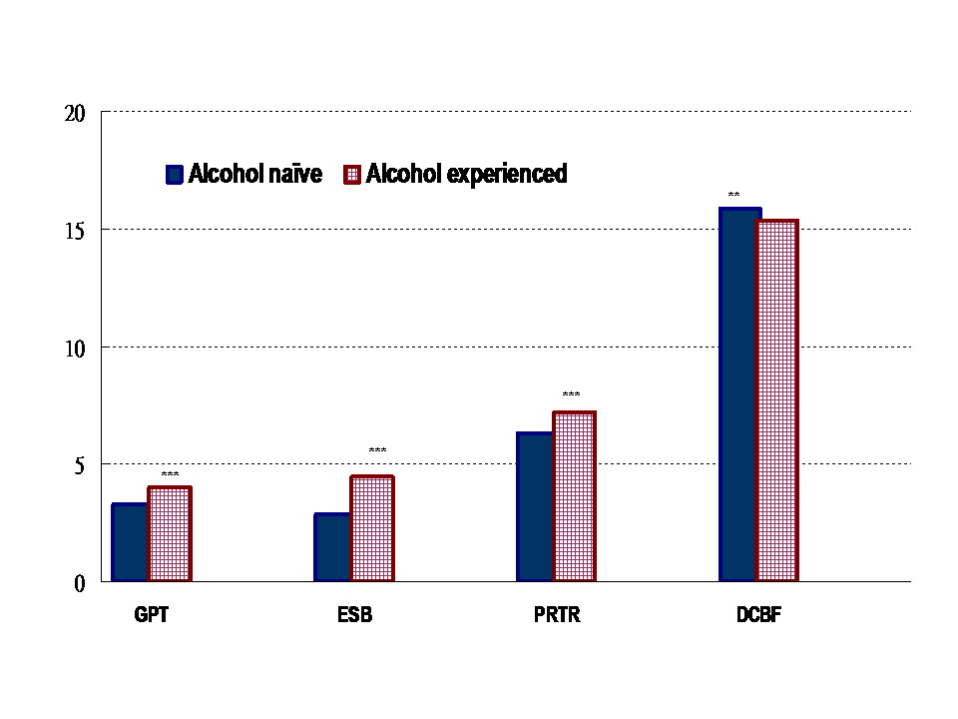
**Estimated averages of alcohol expectation raw scores, by alcohol drinking experience**. Note: Four dimensions of alcohol expectancy: "global positive transformation (GPT),""enhanced social behaviors (ESB),""promoting relaxation or tension reduction (PRTR)," and "deteriorated cognitive and behavioral functions (DCBF)." * p < 0.05; ** p < 0.01; ***p < 0.001 for alcohol experience-related differences in scores.

Alcohol experience-related variation in dimension-specific alcohol expectation was found (Figure 1). Alcohol-experienced children, as depicted by red patterned rectangles, tend to have higher scores on the positive alcohol expectation dimensions, as compared with their alcohol-naïve peers (solid blue rectangles)(all p < 0.001); the effect size, as assessed by Cohen's d, was 0.64 for "ESB," and 0.26 and 0.23 for "GPT" and "PRTR", respectively. In terms of negative alcohol expectations [DCBF], children who had drank alcoholic beverages on at least one occasion tended to have lower scores as compared with their alcohol-naïve counterparts (p < 0.01, effect size = 0.12).

Among children who had never drunk alcohol, being a male was inversely related with negative expectancy toward alcohol (β_wt _= -0.14). As shown in Table 2, having a higher monthly allowance seems associated with higher levels of positive alcohol expectation, although statistical significance appears only for the "promoting relaxation or tension reduction" dimension. Family drinking was found to have a salient relationship with the endorsement of positive alcohol expectancy. While simultaneously taking into account family characteristics, paternal drinking was related with a 0.22 increase in one standardized score for global positive transformation, and the corresponding estimates for "enhanced social behaviors" and "promoting relaxation or tension reduction" were 0.15 and 0.18, respectively; whereas the only significant estimate showing an association with maternal drinking was "enhanced social behaviors" (β_wt _= 0.16). Standardized scores for "enhanced social behaviors" and "promoting relaxation or tension reduction" were also increased for those with siblings who drank. Further efforts were made to explore possible interaction effects. For gender with paternal drinking and maternal drinking, no significant interaction term was found by the p-value of less than 0.05 for four dimensions of alcohol expectancy; for paternal drinking with maternal drinking, the interaction term appeared only significant in negative expectancy (i.e., deteriorated cognitive and behavioral functions [DCBF])(β_wt _= -0.017).

**Table 2:** Estimated relationship between individual and family characteristics with alcohol expectancy in alcohol-naïve children

**Characteristics**^**a**^	Dimension of Alcohol Expectancy			
	
	**GPT β**_**wt **_**(95% CI)**	**ESB β**_**wt **_**(95% CI)**	**PRTR β**_**wt **_**(95% CI)**	**DCBF β**_**wt **_**(95% CI)**
Grade (ref: 4^th^)				
6^th^	0.18 (0.08, 0.30)**	0.08 (-0.04, 0.20)	0.35 (0.22, 0.48)***	0.19 (0.09, 0.30)***
Gender (ref: Female)				
Male	0.07 (-0.03, 0.17)	0.07 (0.01, 0.13)*	0.01 (-0.08, 0.1)	-0.14 (-0.21, -0.07)***
Living with parents (ref: Living with two parents)				
Living with none or one parent	0.06 (-0.08, 0.20)	0.14 (0.01, 0.27)*	0.03 (-0.13, 0.19)	-0.07 (-0.23, -0.10)
Parental employment (ref: None or one)				
Both employed	-0.05 (-0.15, 0.06)	-0.02 (-0.12, 0.09)	-0.05 (-0.16, 0.05)	-0.04 (-0.15, 0.07)
Monthly allowance (NT)(ref: 0)				
1-499	0.09 (-0.01, 0.19)	0.04 (-0.06, 0.14)	0.07 (-0.01, 0.15)	0.08 (-0.01, 0.18)
500 or above	0.13 (-0.01, 0.26)	0.14 (-0.01, 0.29)	0.18 (0.04, 0.32)**	-0.05 (-0.21, 0.11)
After school program attendance (ref: No)				
Yes	0.05 (-0.09, 0.19)	-0.03 (-0.12, 0.07)	0.06 (-0.10, 0.23)	0.17 (0.03, 0.31)*
Paternal drinking (ref: No)				
Yes	0.22 (0.10, 0.33)***	0.15 (0.06, 0.24)**	0.18 (0.04, 0.32)**	0.10 (-0.06, 0.25)
Maternal drinking (ref: No)				
Yes	0.01 (-0.11, 0.12)	0.16 (0.06, 0.27)**	0.02 (-0.15, 0.20)	-0.06 (-0.20, 0.08)
Elder sibling's drinking (ref: No)				
Yes	0.21 (-0.07, 0.49)	0.26 (0.02, 0.50)*	0.25 (0.03, 0.46)*	-0.01 (-0.24, 0.21)

*Notes*: Three scales of positive alcohol expectancy: "global positive transformation (GPT)," "enhanced social behaviors (ESB)," and "promoting relaxation or tension reduction (PRTR)," and one negative alcohol expectancy scale: "deteriorated cognitive and behavioral functions (DCBF)."

a. Estimated regression coefficients were derived from multivariate linear regression (weighted data with Taylor series linearization) with simultaneous adjustment for listed covariates.

*p < 0.05 ** p < 0.01, *** p < 0.001

Moderate drinking-related differences were found in the relationship estimates between family attributes and drinking with the endorsement of alcohol expectancy. After taking into account the same array of individual- and family-level variables listed in Table 2 (not including alcohol-drinking variables), we found that, for the alcohol-experienced children, the observed coefficients associated with maternal drinking were more prominent than those of paternal drinking; the adjusted relationship coefficient of maternal drinking was 0.19 (95% CI: 0.03, 0.36) for "global positive transformation" and 0.33 for "enhanced social behaviors"(95% CI: 0.18, 0.47), and the corresponding estimate for paternal drinking was -0.06 (95% CI: -0.26, 0.15) and -0.15 (95% CI: -0.39, 0.08) (data not shown here). Additionally, receiving a higher allowance was significantly related with greater endorsement of negative alcohol expectancy.

Finally, the adjusted relationship coefficients of individual- and family-variables appear slightly attenuated when personal drinking experiences were taken into account, particularly the estimates of family drinking (see Table 3). The relationship of maternal drinking with GPT and ESB was estimated at 0.17 and 0.23, respectively. As compared with experimental alcohol use, occasional drinking was associated with a 0.54 increase in ESB standardized scores, and a 0.3 reduction in DCBF or negative alcohol expectancy standardized scores. Among the alcohol experienced children, no significant interaction term was found between gender with paternal drinking or maternal drinking and paternal drinking with maternal drinking.

**Table 3:** Estimated relationship between individual and family characteristics with alcohol expectancy in alcohol-experienced children

**Characteristics**^**a**^	Dimension of Alcohol Expectancy			
	
	**GPT β**_**wt **_**(95% CI)**	**ESB β**_**wt **_**(95% CI)**	**PRTR β**_**wt **_**(95% CI)**	**DCBF β**_**wt **_**(95% CI)**
Grade (ref: 4^th^)				
6^th^	0.18 (0.03, 0.33)*	0.13 (-0.03, 0.29)	0.27 (0.15, 0.40)***	0.23 (0.05, 0.40)**
Gender (ref: Female)				
Male	0.18 (-0.01, 0.37)	0.04 (-0.13, 0.20)	-0.004 (-0.22, 0.21)	-0.08 (-0.25, 0.09)
Living with parents (ref: Living with two parents)				
Living with none or one parent	-0.01 (-0.31, 0.29)	0.002 (-0.20, 0.22)	0.08 (-0.15, 0.31)	-0.07 (-0.26, 0.11)
Parental employment (ref: None or one)				
Both employed	-0.01 (-0.20, 0.17)	0.06 (-0.08, 0.21)	0.006 (-0.17, 0.19)	-0.21 (-0.40, -0.01)*
Monthly allowance (NT)(ref: 0)				
1-499	0.08 (-0.13, 0.30)	0.01 (-0.21, 0.23)	0.09 (-0.12, 0.32)	0.25 (0.01, 0.50)*
500 or above	0.12 (-0.08, 0.32)	0.10 (-0.14, 0.34)	0.21 (0.03, 0.38)*	0.34 (0.10, 0.58)**
After school program attendance (ref: No)				
Yes	-0.12 (-0.35, 0.11)	-0.08 (-0.28, 0.12)	-0.10 (-0.30, 0.10)	-0.007 (-0.15, 0.14)
Paternal drinking (ref: No)				
Yes	-0.05 (-0.25, 0.15)	-0.15 (-0.38, 0.07)	0.09 (-0.07, 0.24)	0.05 (-0.16, 0.26)
Maternal drinking (ref: No)				
Yes	0.17 (0.01, 0.34)*	0.23 (0.07, 0.40)**	0.08 (-0.07, 0.23)	-0.01 (-0.19, 0.17)
Elder sibling's drinking (ref: No)				
Yes	0.02 (-0.19, 0.23)	0.27 (0.04, 0.49)*	0.13 (-0.06, 0.32)	0.08 (-0.16, 0.33)
				
Alcohol consumption (ref: Experimental use)				
Occasional use	0.17 (-0.01, 0.34)	0.54 (0.37, 0.72)***	0.10 (-0.07, 0.27)	-0.29 (-0.47, -0.11)**
Age at first alcohol use (ref: 7 or older)				
Younger than 7 year-old	0.09 (-0.09, 0.28)	0.05 (-0.14, 0.23)	0.17 (-0.01, 0.35)	0.11 (-0.07, 0.28)

*Notes*: Three scales of positive alcohol expectancy: "global positive transformation (GPT)," "enhanced social behaviors (ESB)," and "promoting relaxation or tension reduction (PRTR)," and one negative scale: "deteriorated cognitive and behavioral functions (DCBF)."

a. Estimated regression coefficients were derived from multivariate linear regression (weighted data with Taylor series linearization) with simultaneous adjustment for listed covariates.

b. *p < 0.05 ** p < 0.01, *** p < 0.001

## Discussion

In this urban region of Taiwan, over 40% of the elementary-school aged children surveyed had tried more than a sip of alcohol on at least one occasion. Differences in both endorsement and correlates of alcohol expectancy were found by drinking history. Among alcohol-naïve children, older ages and paternal drinking were significantly related with higher levels of positive alcohol expectancy; older ages and being female associated with negative alcohol expectancy. In contrast, for those who had initiated alcohol use, maternal drinking and personal drinking frequency appear more salient in relation to positive alcohol expectancy; younger age, lower monthly allowance, and occasional drinking were strongly related with lower endorsement of negative alcohol expectancy.

Several potential limitations should be considered in interpreting our findings. First, our data were primarily collected from public school-attending young children; therefore, the results may not directly reflect the experiences in private-school attendants (less than 5% in the city of Taipei). Moreover, due to cultural, geographic, and societal differences, it might not be appropriate to generalize these findings outside of Taiwan. Second, selection bias may arise due to differential response rates associated with variables of interests in this study. Further post-hoc analyses have shown that schools with large-scale and convenient transportation (or so-called elite schools) tend to have a lower response rate than the others, suggesting that students from certain higher socioeconomic subgroups may be possibly under-represented in the study population. Third, there are also method limitations of a survey, such as the cross-sectional design and retrospective self-report. The assessment of family drinking relies solely on observation and may be vulnerable to bias if a child's attention to alcohol clues in the environment is associated with his or her alcohol drinking experience or alcohol expectancy. Future studies of multi-wave assessments may help understand complex relationships operating between family context and the endorsement of alcohol expectancy from childhood to adolescence.

Not including a sip of alcohol or alcoholic beverages added in meals, the lifetime prevalence estimate of alcohol drinking in our study was generally higher than those reported in other populations. For example, according to a summary report on children's alcohol use in the US, the lifetime prevalence of "more than a sip of alcohol" was estimated at 10% for 4^th ^graders and 29% for 6^th ^graders in 1999; more recent estimates of "ever having had a drink or having used alcohol" were 18% and 35% among children in Texas in 2004 [39]. The possibility that the differences in lifetime prevalence are attributed to the variation in operational definition cannot be ruled out, but this observation may indeed reflect the diversity in the societal norms or parental attitudes towards underage drinking as well as in the availability of alcohol at home and community [35].

Assessing developmental variation in positive alcohol expectancy among 1^st ^to 5^th ^graders, Miller and colleagues found the endorsement of dimensions of sexual enhancement and global enhancement might increase with age [13]. Our findings also found positive alcohol expectancy to be more salient for "promoting relaxation or tension reduction" and "global positive transformation" among older ages. Noticeably, the age-related increase also appears in the dimension of negative alcohol expectancy "deteriorated cognitive and behavioral functions". Although this observation may be merely a reflection of age (or even cohort) differences rather than developmental variation, still the observed age-related increase in the endorsement of "promoting relaxation," "global positive transformation," and "deteriorated cognitive and behavioral function" suggests that the development of alcohol expectancy may not simply shift from negative to positive. Even within positive alcohol expectation, the development may be dimension-specific or not solely be a linear function of age [14,30,40].

An outreach study of adolescents in Taiwan concluded that higher allowance increased the odds of alcohol, tobacco, and illegal drug use two fold [41]. Similarly, our findings indicate that having more allowance appears slightly related with the endorsement of positive alcohol expectancy (especially "promoting relaxation or tension reduction") and negative alcohol expectation in the alcohol-experienced children, suggesting that monthly allowance, a proxy measure of family socioeconomic status, may influence youngsters' alcohol expectation differentially by dimension. It is possible that children with more disposable money were more likely to engage in leisure activities or scenes where alcoholic beverages are more available to facilitate relaxation (e.g., karaoke or party). This issue should be explored more comprehensively by future multi-wave follow-up data, including the measures of allowance spending and leisure/social activity participation.

Findings from this study suggest that paternal and maternal drinking may have differential relationships with children's drinking expectancy, and the relationship pattern even varies considerably by children's prior drinking experience. The observation may be, in part, the result of father-mother differences in parenting behaviors, alcohol-specific socialization, or alcohol-specific parenting practices [27,28,42]. In addition, we observed dimension-related differences in the linkage between family drinking and the endorsement of alcohol expectancy, suggesting that the emergence of different dimensions of alcohol expectancy in mid- to late childhood may be differentially shaped by the source of social influence (e.g., parents)[30]. For instance, to the extent that a mother is usually the one who serves as the primary caregiver in Taiwan, at really young ages a child's expectancy and alcohol involvement may be influenced more by their mother's drinking behaviors and attitudes toward underage drinking. Given that the onset of alcohol use occurs primarily at home during occasions of entertainment [35], the salience of maternal dinking on the endorsement of "enhancement of social behaviors" may indirectly reflect how children may perceive or relate expected effects of alcohol via the observation of context-dependent drinking behaviors of their mothers.

The correlation pattern between occasional drinking with "enhancement of social behaviors" and "deteriorated cognitive and behavioral function" among the alcohol-experienced children in this study was consistent with prior studies on the population aged 12 years or above [25,43]. The salient effects of alcohol drinking with enhanced social behaviors may be partially explained by development-related variation in response to ethanol-induced social behavior. For instance, some recent evidence from animal models with developmental comparison supported the idea that adolescents were more sensitive to ethanol-induced social facilitation and less sensitive to ethanol-induced social inhibition as compared to their adult counterparts [44,45]. The observation may also be a reflection of a drinking culture in the study population in which alcoholic beverages are usually served as a social facilitator to promote interaction or cheerfulness, particularly in the context of family, social, and entertainment occasions [35]. Although a temporal relationship may not be established in this cross-sectional study between alcohol drinking frequency and the endorsement of alcohol expectancy, it is very possible that a reciprocal relationship may operate between drinking and the development of alcohol expectancy [37], and the reciprocal process may operate differentially by dimension in childhood.

## Conclusions

In sum, our study indicates the significant role of family drinking on positive alcohol expectancy in one's early life and alcohol drinking-related differences in the correlates for the endorsement of alcohol expectancy in childhood. Findings from this study are particularly noteworthy because they were obtained from a population-based study with a focus on primary school-aged children. Individual tailored strategies that address family influences on alcohol expectancy may be needed in prevention programs targeting drinking behaviors in children. Expectation, especially positive effects, toward alcohol should be considered as a potential marker to shape or monitor in order to delay early initiation or experimentation of alcohol [9,46]. Additional research is warranted to understand possible mediational or moderational effects of alcohol outcome expectancy on the pathways linking social (e.g., family drinking and socioeconomic status) and individual factors with alcohol initiation and regular drinking in early childhood.

## Competing interests

The authors declare that they have no competing interests.

## Authors' contributions

CYC, WJC, and KML developed the study and CYL carried out sampling. CYC conducted the analyses; CYC and CLS drafted the article. KHC helped the implementation of data collection and management. All of the authors contributed to the interpretation of results.

## Pre-publication history

The pre-publication history for this paper can be accessed here:

http://www.biomedcentral.com/1471-2458/11/87/prepub
